# Do Drugs Used in Obstetric Anesthesia Interfere with Early Breastfeeding? Characteristics of The Pharmacodynamic and Pharmacokinetic Properties of Certain Drugs. Part 2

**DOI:** 10.34763/devperiodmed.20192304.233244

**Published:** 2021-01-29

**Authors:** Maria Wilińska, Wojciech Walas, Ewa Kamińska, Małgorzata Malec Milewska, Agnieszka Sękowska, Joanna Stankiewicz, Ewa Helwich

**Affiliations:** 1 Department for Neonatology, Prof. W Orlowski Independent Public Clinical Hospital, Centre of Postgraduate Medical Education, Warsaw, Poland; 2 Department for Anesthesiology and Intensive Care Unit for Children and Neonates, Clinical University Hospital, Opole, Poland; 3 Department of Pharmacology, Institute of Mother and Child, Warsaw, Poland; 4 Department for Anesthesiology and Intensive Care, Prof. W Orlowski Independent Public Clinical Hospital, Centre of Postgraduate Medical Education, Warsaw, Poland; 5 Anaesthesiology and Intensive Care Clinic, Institute of Mother and Child, Warsaw, Poland; 6 Department for Neonatology and Neonatal Intensive Care, Institute of Mother and Child, Warsaw, Poland

**Keywords:** cesarean section, hypnotic drugs, volatile anesthetics, muscle relaxants, analgesics, breastfeeding, cięcie cesarskie, leki nasenne, lotne środki znieczulenia ogólnego, leki zwiotczające, leki przeciwbólowe, karmienie piersią

## Abstract

Cesarean section requires the administration of drugs that shou/d be limited to specific medicolindications.

It is important **to** remember that most of the available and currently administered anesthetics can affect the fetus and the newborn. In obstetric anesthesia, only such medication that demonstrates a beneficial pharmacokinetic profile and maximum effectiveness should be administered.

In this article, the authors reviewed the pharmacodynamic and pharmacokinetic properties of the drugs used during anesthesia in obstetric procedures. The analysis of the influence of these drugs on the clinical condition of the newborn at birth and during breastfeeding was also presented. Drug safety was determined in the aspect of lactation and natura/ feeding.

## Streszczenie

Cięcie cesarskie, które wiąże się z koniecznością podawania leków, powinno być ograniczone do ścisłych wskazań medycznych. Należy mieć na uwadze, że większość dostępnych i stosowanych obecnie leków znieczulających może wywierać wpływ na płód i noworodka. Do znieczulenia w położnictwie należy dobierać leki o korzystnym profilu farmakokinetycznym, z zachowaniem optymalnej skuteczności.

W niniejszym artykule dokonano przeglądu właściwości farmakodynamicznych i farmakokinetycznych leków używanych do znieczulenia w położnictwie. Przedstawiono także analizę wpływu tych leków na stan kliniczny noworodka przy urodzeniu i podczas karmienia piersią. Określono bezpieczeństwo leków w aspekcie laktacji i karmienia naturalnego.

## Introduction

All medications used to anesthetize a woman during labor permeate into the blood of the baby as well as into the mother s milk. The degree of the transfer and its influence on the fetus and the newborn are varied.

In the following paper, we focused on the pharmacological properties of the different medications and also on their pharmacokinetic and pharmacodynamic characteristics. The anesthesiological aspects in obstetric drugs have been presented briefly, as we concluded that a thorough presentation goes beyond the scope of this paper.

## The characteristics of the following medications

In the writing of this paper we used the Summary Product Characteristics of the medicinal products. Information on drug safety during pregnancy (the categories ) contains data according to the Food and Drug Administration (FDA) [1]; drug lactation categories were used according to Hale (2019) [2].

### Intravenous anesthetics

Intravenous anesthetics are used in order to begin anesthesia and sustain it.

### Propofol (pregnancy: B, lactation: L2)

Propofol is a hypnotic drug that works quickly and short-term. It leads to the loss of consciousness in 25-40 seconds and sustains this state for 4.4 -8 minutes. In a subhypnotic concentration, it induces a calming and amnestic effect. It binds to plasma proteins to an extent of 98%.

Propofol is used in the induction of anesthesia and in sustaining it as an agent for total intravenous anesthesia (TIVA). In principle, it is administered in continuous infusion with an opioid.

The most significant adverse effects include a depressive effect on the circulatory system, which manifests in the decrease of blood pressure and in respiratory depression.

In a pregnancy that is carried to term, after permeating through the placenta, the concentration of propofol in cord blood is 70% of that of the mother. When used for cesarean section it does not generate a negative effect on the health conditions of the fetus or the newborn after birth. The weakening of the neurobehavioral state of the fetus as described on the Early Neonatal Neurobehavioral Status (ENNS) scale occurred rarely and was temporary and mild [3, 4, 5]. Propofol has been proven to be superior to thiopental in cesarean sections [5].

The concentration of propofol used in anesthesia in cesarean section in mother s milk is small. In a 24-hour process of collecting colostrum, the concentration of the drug was 0.027% of the dose given to the mother [6].

Even when taking into account the total absorption of the medication by the newborns gastrointestinal tract, this amount is insignificant when compared to transplacental transfer.

During breastfeeding, the newborn receives through milk no more than 1% of the medication given to the mother [2]. The medication is quickly eliminated from the newborns circulatory system.

MW 178 g/mol, pKa 1.1, Vd 4.6 L/kg, EPB 98%, t0,5 distrib. 2-4 minutes; t0,5 redistrib. 30-60 minutes (IV bolus); terminal elimin. t0,513-44 h (IV infusion), M/P ratio approx. 1, RID 4.44% [2, 4, 6, 7].

RECOMMENDATION: Propofol used in obstetrics is a safe drug regarding its influence on the fetus, the status of the newborn after birth, and also the aspects of natural feeding. It is recommended, however, to observe newborns directly after birth in terms of any adverse impact on breathing and decreased activity during feeding.

### Thiopental (pregnancy: C, lactation: L3)

Thiopental is a derivative of sulphur barbituric acid that works ultra-rapidly and short-term. Its function involves suppressing the reticular formation of neurons and decreasing the duration of the REM sleep phase. The speed of its effect on the central nervous system (CNS) is connected to the easiness with which the medication dissolves in fats.

After intravenous injection, thiopental reaches the brain in no longer than 30 seconds, and causes loss of consciousness that lasts 5-10 minutes. After 5 minutes, the concentration of the drug in the brain already decreases to half of its initial value. The rapid phase of thiopental distribution is 8.5 minutes and is the cause of the short duration of the medication.

The drugs redistribution to tissues with a poor blood supply is a process that progresses slowly. The balance between the muscles and the skin is reached only after 1-30 minutes, and for adipose tissue it is achieved only after 1 hour. This slow phase of distribution takes 62.7 minutes and the volume of distribution is 2.5 L/kg. In pregnant women at term, the Vd may increase to 4.1 L/kg [8].

Thiopental is used in the induction of anesthesia as an anesthetic and also to control convulsions. It is particularly recommended for the induction of anesthesia in people suffering from epilepsy.

The most significant adverse effects include a depressive effect on the circulatory system, which manifests in the decrease of blood pressure. Additionally, the drug causes respiratory depression, contraction of the glottis and the bronchial tubes. It is a strong inductor of liver enzymes, and as such, accelerates the metabolism of other drugs and endogenous substances, such as steroid hormones and bile acids.

Thiopental administered to a pregnant woman is detected in the fetal blood as soon as one minute from the time of the injection in a concentration not much lower than that of the mother s. However, the concentration in the CNS of the fetus is small and short-term, because of the quick elimination of the drug. Therefore, when it is used in anesthesia in standard doses, it does not have a significant effect on the fetus and on the state of the newborn after birth. Higher doses may lead to respiratory depression in the newborn.

Thiopental has elimination t0 ,5of 4-12 h, but it is much longer in pregnant women at term: 26-28 h [8]. The M/P ratio is about 0.30 for colostrum, and 0.40 for mature milk. The maximum concentration of the drug in milk is 0.9 mcg/ml. A neonate being fed only with mother s milk shows a concentration of the drug in the serum of about 0.135 mg/kg, which is about 2-3% of the mother s dose when calculated according to body weight in kilograms. In other words, during the first feeding it is less than 0.1% of the dose. We should not be afraid that a newborn fed only with breastmilk would have toxic symptoms even considering a longer time of drug elimination by the baby, when we take into account the decreasing concentration of the medicine in the womans blood and consequently in her milk.

MW 264 g/mol, pKa 7.6, t_max_ 1-2 minutes, V_d_ 1.4-2.5 L/kg, EPB 60-96%, t_0_,5 distrib. 8.5 minutes; t0,5 redistrib. 63 minutes; t_0,5_ elimin. 4-12 h (mean 9 h), PHL 15 h, M/P 0,3-0,4, RID 1.77-5.94% [2, 9].

RECOMMENDATION: Thiopental used in obstetrics is a safe drug regarding its influence on the fetus, the status of the newborn after birth, and breastfeeding. It is recommended, however, to observe newborns directly after birth for excessive sedation, decreased activity during sucking, and a potentially undesirable effect on breathing.

### Etomidate (pregnancy: C, lactation: no data available)

Etomidate used in the induction of anesthesia is a hypnotic drug that works quickly and short-term. It affects the reticular formation in the brain stem in a depressive manner, probably by stimulating the gammaaminobutyric acid system (GABA).

After intravenous injection, it causes loss of consciousness within 15-45 seconds, which usually lasts about 3-12 minutes. This depends on the dose and is prolonged with its increase. Within one minute of injection, etomidate reaches the maximum concentration in the blood. About 75% of the drug binds to plasma proteins. In organs which are richly supplied with blood, including the CNS, the drug reaches the same concentration as in the blood. As a result of such a rapid redistribution, the concentration of the drug in the plasma decreases already after one minute of intake. In adipose tissues, the maximum concentration is reached after 10-25 minutes.

Etomidate is used in the induction of anesthesia, particularly in high-risk circulatory system patients. It has a large margin of safety as a result of its low impact on the respiratory and circulatory systems. However, in cesarean section etomidate has a limited application as a result of myoclonus and dyskinesia, which for some patients may be present during the phase of falling asleep. An opioid administered early on prevents such reactions.

Etomidate can be used during pregnancy to anesthetize the patient. In the case of using it as an anesthetic during cesarean section, the newborn may show characteristics of respiratory depression.

The degree to which the drug permeates into the blood and the way newborns tolerate it have been studied briefly. So far, there have been no reports of adverse effects in newborns being breastfed by mother’s who have been given etomidate in anesthesia. Etomidate has not been detected in any colostrum tests after 4 hours from the time of its application in anesthesia [9].

MW 244 g/mol, pKa 4.24, tmax 1 minute, Vd 2-4.5 L/ kg, EPB 75%, t0,5 distrib. 2,7 minutes; t0,5 redistrib. 29 minutes; t0,5 elimin. 2.9-5.3 h [9, 10].

RECOMMENDATION: When the mother regains full consciousness after anesthesia, the newborn can be latched-on. No described pharmacodynamic characteristics of the drug or clinical observations indicate the need to delay or stop breastfeeding.

### Ketamine (pregnancy: B, lactation: L3)

Ketamine is an anesthetic that works rapidly with a hypnotic and analgesic effect. It is the only anesthetic that stimulates the circulatory system accelerating the heart function and simultaneously increasing blood pressure. Ketamine has a strong painkilling effect which occurs 45-50 seconds after intravenous injection and is sustained for about 10-20 minutes. The drug does not cause respiratory depression.

The degree to which it is bound to proteins is small: 10-30%. Because of its good solubility in lipids, ketamine quickly transfers into the brain after intravenous injection. About 10 minutes after administering the drug, its concentration in the brain is slight, whereas its concentration in other tissues which are heavily supplied with blood is 70%.

The safety of ketamine during pregnancy has notbeen determined. In research on animals, an increased risk in congenital developmental defects has not been observed, even if the doses exceeded 10-25 times those

used in humans. However, in microscopic preparations of various tissues, numerous degenerative changes have been reported. The negative effect of ketamine on the development of the human fetus has not been reported. It is not recommended to use ketamine during pregnancy, except to administer the drug during cesarean section or in the course of natural birth.

Ketamine permeates through the placenta. It increases the tension of the uterine muscle and intensifies the strength and frequency of uterine contractions. The effect depends on the dose. After administering a dose larger than 2 mg/kg, a considerable increase of tension in the uterus has been observed. High doses affect the fetus in a depressive way, causing observable disturbances in the biophysical monitoring of the baby.

Ketamine used in anesthesia for cesarean section may cause behavioral abnormalities of the woman and the baby. This is a significant limitation in using the drug. There are observable abnormalities in the process of the baby’s sucking [11]. The negative influence on the baby has not been noticed if the time between administering the drug to induce anesthesia and exiting anesthesia does not exceed 10 and 1.5 minutes, respectively [12].

There is no research describing the tolerance for and safety of breastfeeding. At the same time, no significant adverse effects of ketamine in a newborn who has been breastfed, have been reported. Due to the lack of data concerning the safety of using this drug, its use by women who are breastfeeding is not recommended.

MW 274 g/mol, pKa 7.5, tmax 1 minute (IV), BA oral 20-30%, Vd 2.4 L/kg, EPB 10-30%, t0,5 distrib. 11-16 minutes, t0,5 elimin. 2.5 h, [2, 13].

RECOMMENDATION: It does not seem that ketamine can be recommended for use during pregnancy. The exception is anesthesia during cesarean section for a patient in shock caused by hemorrhage of the genital tract, with the exception of uterine rupture and a prolapsed umbilical cord. The monitoring of the newborn includes observation for lethargy, oversensitivity to stimuli, and weak sucking.

## Volatile agents (inhalational anesthetics) for general anesthesia

Volatile agents of general anesthesia are inhalational anesthetics that include nitrous oxide and the following anesthetic pairs: halothane, enflurane, isoflurane, sevoflurane, and desflurane. They are used for the induction and sustenance of anesthesia by inhalation.

In the case of inhalational anesthetics, typical pharmacokinetic parameters do not play such a role as in the case of intravenous anesthetics. When using volatile agents, the blood-gas partition coefficient and brain-blood partition coefficient are important.

The blood-gas partition coefficient is the most important factor in determining the speed of induction and recovery. A higher value of this parameter correlates with higher blood solubility and a slower induction rate. A lower value corresponds with a faster induction rate. The higher the brain-blood partition coefficient, the higher the solubility of an agent in brain tissue; the lower this value, the faster the effect ceases [14].

### Desflurane (pregnancy: B, lactation: no data available)

Among all the inhalational anesthetics, desflurane is characterized as the most controllable one. It causes the quickest induction of anesthesia, enables the fastest awakening of the patient, and makes the depth of anesthesia easiest to control. It mainly affects the circulatory and respiratory systems in a depressive way. The disadvantage of desflurane is its irritating effect on the upper respiratory tract, limiting its use in the induction of anesthesia.

There are no published reports of the use of desflurane during breastfeeding. Because the half-life of desflurane in the mother s serum is short, it does not seem that the agent permeates into the mother’s blood. There is no need to delay or postpone breastfeeding. Breastfeeding can be started as soon as the mother regains consciousness after general anesthesia.

Desflurane belongs to the standard agents used in anesthesia during pregnancy or cesarean section. Newborns should be monitored in regard to respiratory and circulatory strength, as well as hyperbilirubinemia.

Blood-gas partition coefficient 0.42; brain-blood partition coefficient 1.3 [15].

### Sevoflurane (pregnancy: B, lactation: L3)

It is characterized by lower steering than desflurane. It does not irritate the upper respiratory tract and thanks to that it is applicable for the induction of anesthesia by inhalation. The effect on the circulatory system and respiratory system of the anesthetized patient is close to that of desflurane.

Blood-gas partition coefficient 0.69; brain-blood partition coefficient 1.7 [15].

Inhalational halogen agents are standard preparations used for anesthesia in obstetrics. The newborn should be observed in terms of respiratory and circulatory stability. This should be done as soon as the mother can start breastfeeding after anesthesia. No pharmacokinetic parameters of these agents used for anesthesia or clinical observation justify the need to delay or stop breastfeeding. This recommendation also refers to anesthesia during cesarean section. The transfer of the agents through the placenta is higher than their transfer to mother’s milk. There is a slightly increased risk of hyperbilirubinemia [16].

RECOMMENDATION: During pregnancy, desflurane and sevoflurane should only be used in cases of utmost urgency. They can be used in anesthesia for cesarean section. High concentrations and prolonged time of use should be avoided, because the risk of excessive relaxation of the uterus muscle of the mother and respiratory depression of the newborn increases. The agents cross the placental membrane. As they are released into the mother’s milk, reaching only infinitesimal concentrations, they should be used with caution during breastfeeding.

### Nitrous oxide (pregnancy: no data available, lactation: L3)

Laughing gas. Nitrous oxide is an inorganic, colorless, odorless and tasteless gas that is usually used to supplement general anesthesia. Its transport in the plasma takes place only in the form of a physically dissolved gas. Its solubility in the blood is similar to desflurane, however its solubility in the brain and other organs is lower than any other inhalational anesthetics. Therefore, the pressure of the gas in the brain rapidly increases during its fast introduction to anesthesia and the effects happen accordingly quickly. After it stops being administered, the gas is immediately eliminated from the blood through the lungs and the patient wakes up quickly.

Nitrous oxide is a weak anesthetic, therefore it is mainly used as a supplement for other anesthetics, making it possible to decrease their dosage.

Nitrous oxide quickly transfers through the placenta and after 3-4 minutes the mother-fetus concentration gradient is 0.8. It transfers into the fetal tissues equally fast. During protraction of the anesthesia, the gradient of concentration between the blood in the umbilical artery and the blood in the umbilical vein increases. After 15-36 minutes, the concentration balance of about 90% is established.

The prolonged use of nitrous oxide (more than 15-17 minutes) is connected with the increase of its depressive influence on the newborn. There is no data concerning the transfer of nitrous oxide into human milk.

Blood-gas partition coefficient 0.47; brain-blood partition coefficient 1.1 [15].

RECOMMENDATION: Nitrous oxide is a safe drug for the newborn. However, the neonatologist has to know the drug has been applied in the woman, so that the efficiency of the newborns respiratory system after birth can be controlled.

### Opioids

Out of numerous opioids, fentanyl and sufentanil are most commonly used in obstetric anesthesia. Other opioids, such as morphine, alfentanil, remifentanil, are used in anesthesia mostly to take out the remains of the placenta, and possibly for surgery conducted after birth.

Opioids show a strong analgetic effect, moreover, they cause somnolent and euphoric effects.

In obstetrics, the use of opioids in general anesthesia is limited. After intravenous injection, they permeate through the placenta quickly and to a considerable extent. Considering the depressive effect on the fetus and the newborn, especially with a tendency towards apnea, the use of opioids in anesthesia should be limited only to the introductory stage of dilation. In anesthesia for cesarean section, they should not be used before the clamping of the umbilical cord. In regional anesthesia for natural birth, they are used with local anesthetics.

### Fentanyl (pregnancy: C, lactation: L2)

Fentanyl is a medication, which is strongly lipophilic and quickly permeates through the brain-blood barrier. Within 3-5 minutes it reaches maximum concentration. It spreads quickly in tissues and its concentration in the plasma decreases rapidly. The high gradient established in this way favors the outflow of fentanyl from the brain and causes a short-term effect.

The maximum effect occurs 5-8 minutes after injection and in a standard dose is sustained for 20-30 minutes. Using fentanyl in general anesthesia in obstetrics should be limited to the introductory phase dilation, due to the danger of respiratory depression for the newborn. When used cautiously in epidural anesthesia during labor, the risk of CNS and respiratory depression is infinitesimal both for the mother and the newborn. This course of action is considered safe. There is a slightly higher risk connected with administering the drug into the subarachnoid space.

In the 24-hour procedure of collecting milk, the concentration of fentanyl was established as 0.033% of that of the mother’s blood concentration [6]. In practice, less than 1 % of the dose administered to the mother permeates into the blood. The highest concentration in milk is observed about 45 minutes after giving the mother an intravenous injection of fentanyl. Afterwards, the concentration quickly decreases, and after 10 hours it is untraceable. The assimilability of fentanyl from the newborns digestive tract is infinitesimal, which is why the drug can be safely used to anesthetize breastfeeding mother’s and women during labor. The short half-life and quick elimination of the medicine from circulation lead to low permeation into mother’s milk and an irrelevant clinical influence on the baby.

MW 336 g/mol, pKa 8.4, tmax 3-5 minutes (IV); 7-8 minutes (IN); BA oral 50-75%, Vd 4-6 L/kg, EPB 80-85%, t0,5 elim. 2-4 h (IV), PHL 317-1266 minutes (newborns), RID 2.9-5% [2, 4].

### Sufentanil (pregnancy: C, lactation: L4)

Sufentanil is a very strong opioid. It is 7-10 times stronger and also faster than fentanyl. Moreover, it is more lipophilic than fentanyl. It binds harder to opioid receptors. Fentanyl distributes quickly in the peripheral lymphoid organs. Because of its high degree of ionization and a significant degree of binding to plasma protein, its volume of distribution is lower. The half-life of sufentanil is shorter than that of fentanyl. The maximum effect of the drug occurs about 2-4 minutes after injection and in a standard dose lasts 30 minutes.

The use of sufentanil in general obstetrics anesthesia should be limited to the introductory phase of dilation, because of the risk of respiratory depression in the newborn. When used cautiously in epidural anesthesia during labor, the risk of CNS and respiratory depression is infinitesimal for the mother as well as the newborn. There is a slightly higher risk connected with administering the drug into the subarachnoid space.

The medication quickly permeates through the placenta. The ratio of sufentanil concentration in the unborn baby’s vein blood to the mother’s vein blood is 0.8:1. Using it before the clamping of the umbilical cord during pregnancy and the procedure of cesarean section is contraindicated. Sufentanil can exert an inhibitory effect on the newborns respiratory system. In such a situation, on the basis of the pharmacokinetic data we should assume that breastfeeding may start 24 hours after general anesthesia.

Sufentanil permeates into human milk. Caution should be advised when administering sufentanil to a breastfeeding woman.

MW 386 g/mol, pKa 8.01, Vd2.9 L/kg, EPB 92%, t0,5 distrib. 1.4 minutes; t0,5 redistrib. 17.7 minutes; t0,5 elim. 164 minutes [17].

### Alfentanil (pregnancy: C, lactation: L2)

Alfentanil is an opioid that works more weakly than fentanyl, with a faster and shorter effect. The maximum painkilling and breathing inhibitory effects occur about one minute after intravenous injection, and the minimal time of its effect is 11 minutes. Alfentanil is less lipophilic than fentanyl, it binds less to adipose tissues and muscles. The ionized fraction constitutes only 11%. It is 92% bound to plasma protein.

The elimination half-life of alfentanil is shorter than that of fentanyl and sufentanil. It is shorter in children (4255 minutes) than in adults (1-2 h) [17]. In the umbilical cord blood, the drug reaches a concentration of 30% of that in the mother’s blood.

The transfer into the milk of alfentanil administered in subdural analgesia during a CS anesthesia is low. Up to the third day after surgery, the concentration is 0.05 mcg/L, and it does not cause neurological adverse effects.

The potential effect of alfentanil on the newborn includes respiratory depression and hypertension.

MW 417 g/mol, pKa 6.5, tmax immediate, BA oral 43%, Vd 0.3-1.0 L/kg, EPB 92% t0,5 elim. 1-2 h, PHL 42-55 minutes, RID 0.26-0.4% [2, 17].

### Morphine (pregnancy: C, lactation: L3)

Morphine is currently used less frequently in general anesthesia for cesarean section and in regional anesthesia in obstetrics. Its use during labor would entail a risk of respiratory depression for the newborn. It is used in mitigation of the pain when taking the remains of the placenta out of the uterus, or during minor surgeries after birth.

The concentration of morphine and its metabolite, morphine -6- glucuronide, in the colostrum of mother’s who were administered morphine in the analgesia of labor in cesarean section is very low [11, 18]. The highest concentration of morphine in milk after administering it in subdural anesthesia occurred in 30 minutes and was 82 mcg/L. After 4 hours since its administration the concentration of morphine in milk is untraceable.

There have been no reports of harmful effects of morphine used in mothers who were breastfeeding their newborns [19, 20].

Morphine administered through an infusion pump for the mitigation of pain after CS throughout 12-48 hours reaches a concentration of 50-60 mcg/L in milk.

Newborns that are tested in the third day of life do not demonstrate any change in a neurological examination. The bioavailability of morphine in a newborn after oral administration is very small (26%) and that is why the drug is easily tolerated by a baby being breastfed.

Newborns have an extended time of morphine elimination and a lowered clearance of the medication when compared to infants. After 2 months, newborns reach values that are similar to those of adults. Morphine is one of the safest drugs of this group during lactation, because the absorption from the gastrointestinal tract is very small. A prolonged administration of morphine in high doses can cause excessive sedation and respiratory disorders in the newborn.

MW 285 g/mol, pKa 8.1, tmax 0.5-lh, Vd 2-5 h, EPB 35%, t0,5 elim. 3-4 h, PHL 6-12 h (preterm infants), BA oral 26%, M/P 1.1-3.6, RID 9.09-35% [2, 10, 18, 21].

### Remifentanil (pregnancy: C, lactation: L3)

Thanks to a structure different from that of other opioids, remifentanil is characterized with a therapy steerability that can be compared to inhalational anesthetics. This results from a rapid dissolution of the drug in the organism. That is why remifentanil is suited for both the introduction and the sustenance of anesthesia, particularly as a component of TIVA.

The value of pKa - 7.07 - indicates a possibility of re-entry from the milk into the plasma, according to the current difference of concentration. It is a safety mechanism that prevents the gathering of the medication in the lactiferous alveoli. As far as the aspect of natural safety of breastfeeding is concerned, an additional beneficial parameter is its molecular mass. For remifentanil, it is 412.

After intravenous injection, the maximum concentration is reached in the place of its effect within 1-1.5 minutes. After 6 minutes the concentration is only about 20%. The time that the maximum effect occurs after intravenous injection is 1.5-2 minutes, and the minimal time of its effect is 10 minutes. The transfer of the medication from the mother’s blood into the lactiferous alveoli in the mammary gland is insignificant.

Remifentanil is 70% bound to plasma protein, which is less than other opioids. This means that it binds heavily with the mother’s plasma albumins and as a consequence the transfer into the mother’s milk is weak. Its solubility in lipids is also low. The partial time of determining the balance between the blood and the brain is 1-1.5 minutes for remifentanil, similarly as for alfentanil.

The bioavailability barrier for the newborn is its category of absorption from the gastrointestinal tract, which in the case of remifentanil is categorized as poor.

The volume of distribution of remifentanil is 0.1 L/ kg. This means that the medication transfers into tissues other than the mother’s blood to a minimal degree, and as a consequence, its presence in the organism is very short. Additionally, its very short value of half-life means that the medication will be quickly eliminated from the human organism.

Because of this kinetics, its short half-life, and as a result of its poor bioavailability, it is unlikely that the medication will attain clinically substantial concentration in mother’s milk [22, 23].

MW 412 g/mol, pKa 7.07, Vd 0.1 L/kg, EPB 70%, t0,5 distrib. 3-6 minutes; t0,5 elim. 10-20 minutes, BA oral - weak [2].

RECOMMENDATION: Opioid painkiller medication used short-term before the baby’s birth may be the cause of respiratory disorders for newborns after they are pulled out. These medications can be used during breastfeeding, but only for a short time. Because of a depressive effect on breathing, the newborns of these mothers should be observed. An increased risk of apnea has been reported.

### Muscle relaxants

Muscle relaxant agents are medications that cause reversible flaccid paralysis of skeletal muscles. In general anesthesia for cesarean section it is mandatory to get quick and certain muscle relaxation before intubation. Suxamethonium is used for this purpose, which binds with cholinergic receptors causing their long-term depolarization. When there are absolute contraindications for its use, rocuronium is applied. In obstetric anesthesia, cisatracurium is also used, which is characterized by a process of dissolution and elimination independent from the liver and kidneys.

### Suxamethonium (pregnancy: C, lactation: L1)

Suxamethonium (also known as succinylcholine) is a medication that works quickly and short-term. It is used in the induction of general anesthesia, before the

intubation of the trachea in situations requiring fast and certain muscle relaxation, such as anesthesia for cesarean section. It stimulates all cholinergic autonomic nerve lines, such as nicotine receptors in sympathetic and parasympathetic ganglia, and additionally cholinergic muscular receptors in the sinoatrial node of the heart. Because of this, suxamethonium can cause various adverse effects, including abnormal heart rhythm, apart from its effect of relaxation of the skeletal muscles.

The drug’s dissolution takes place in the process of plasma and liver pseudocholinesterase activity. The hydrolytic capability of pseudocholinesterase is so great that only a minor part of the drug injected intravenously reaches the neuromuscular junction. After a typical intubation dose, the maximum relaxation level is reached within 60-90 seconds and this effect lasts only 5-10 minutes. The full strength of the muscles returns after 12-15 minutes.

Thanks to its low solubility in fats and its ionized particle characteristics, the drug does not permeate through the placenta. When considering its use, the risk of apnea has to be taken into account (it is assessed that this effect can occur after administering a dose >1 mg/kg). The drug can be used during the period of breastfeeding.

### Rocuronium (pregnancy: C, lactation: no data available)

Rocuronium is a non-depolarizing medication of muscle relaxation which is characterized by the fastest effects of all the medications in this group. This is a factor which results in its usefulness in introduction to general anesthesia in case of contraindications for the use of suxamethonium. The maximum effect that enables the intubation of the trachea occurs as soon as 1-2 minutes after intravenous injection. The time of its effects is moderately long and is about 35 minutes. The ratio of returning to norm is 12-15 minutes. Its half-life is 84-131 minutes.

The few adverse effects are clinically insignificant.

Rocuronium bromide transfers through the placenta to a very small degree and it does not cause clinically significant adverse effects in the newborn. When administered as an introduction to anesthesia, it does not influence the clinical state of the baby after birth according to the Apgar scale. There is no data about the transfer of rocuronium into milk of breastfeeding mothers. Research conducted on animals has shown the presence of a slight amount of rocuronium bromide in females’ milk.

For women who are breastfeeding, this medication should only be used, when the doctor’s opinion states that the advantages outweigh the disadvantages. After administering a single dose, it is recommended breastfeeding should be stopped for the time corresponding to five half-lives, which is about 6 hours.

### Cisatracurium (pregnancy: B, lactation: no data available)

Cisatracurium is a non-depolarizing medication of muscle relaxation, which similarly to atracurium and in contrast to other medications from this group is characterized with a peculiar dissolution, which is independent from the function of the liver and kidneys. After intravenous injection of a standard dose, the effect occurs after 2-3 minutes and lasts for about 45 minutes. There is a possibility of slight adverse effects on the circulatory system but they are clinically insignificant.

A unique way of elimination determines the usefulness of cisatracurium in the anesthesia of patients with severe dysfunctions of the liver and kidneys.

The concentration of muscle relaxing medications administered to the women in a pregnancy carried to term measured in the blood of the umbilical cord is 10-20% of the mother’s concentration. This means that there is significant transplacental transport. However, using this group of medications during labor is highly tolerated by newborns. Because of the high ionization of these drugs and their limited solubility in fats, muscle relaxing medications in contrast to anesthetic medications administered in a general and local way have a hard time getting through the blood-cerebrospinal fluid barrier. This explains the lack of myorelaxation effect in the fetus [22, 23].

RECOMMENDATION: Skeletal muscle relaxants can be used as part of an anesthetic set during pregnancy and labor.

MW 929 g/mol (1243 g/mol as besylate), pKa 19.02 (as besylate), Vd 0.12-0.16 L/kg, t0,5 22-29 minutes [24].

### Antiemetics and medications reducing gastric pH

#### Metoclopramide (pregnancy: B, lactation: L2)

Metoclopramide is an antiemetic medication with a central effect, which blocks the dopamine receptor and lowers the sensitivity of lumbar splanchnic nerves that transfer the impulses from the gastrointestinal tract to the emetic center in the brain. The circumferic effect includes stimulating the peristalsis of the upper part of the gastrointestinal tract and increasing the tension in the lower sphincter of the gullet. During breastfeeding, metoclopramide is sometimes used in order to stimulate the release of prolactin by the pituitary gland. There is a noticeable increase of prolactin concentration and an increase in the volume of the milk that is generated. Women with a high concentration of endogenous prolactin do not react with an increase of lactation after having metoclopramide administered.

Metoclopramide reaches the highest concentration in

mother’s milk 2-3 hours after administration. The drug does not accumulate in the baby’s organism. There is no observable adverse effect.

There is a need to pay attention to sleepiness, loose stools, and extrapyramidal symptoms in the baby being breastfed.

MW 300 g/mol, pKa: 9.7; pKa2 0.42, t 1-2 h, Vd 0.1 L/kg, EPB 30%, t0,5 5-6 h, BA oral 30-100%, M/P 0.5-4.06, RID 4.7-14.3% [2].

#### Ondansetron (pregnancy: B, lactation: L2)

A selective antagonist of serotonin (5-HT3), which is used as an antiemetic drug in treating patients suffering from nausea and vomiting. It very rarely causes weakness, decreased activity, excessive sedation and impaired sucking in newborns. These symptoms do not require treatment. The half-time of the medication is 2-3 hours. There is no data concerning the transfer into human milk.

MW 293 g/mol, pKa 7.4, tmax 10 minutes (IV); 40 minutes (IM); 0.5-2 h (PO), BA oral 56-66%, Vd 2.3 L/kg, EPB 70-76%, t_0,5_ 3-4 h, PHL 6.7 h (infants 1-4 months); 2.9 h (> 4 months) [2, 25].

#### Ranitidine (pregnancy: B, lactation: L2)

The blocker of H2 receptors used to decrease the secretion of hydrochloric acid by the stomach lining. It transfers into mother’s milk in quite a high dose. The bioavailability of the drug for a baby who is being breastfed is not great. The concentration in the newborns’ blood is 10 times lower than the therapeutic doses used for the treatment of babies. Single doses used in women giving birth are well tolerated by the mother and baby and there are no contraindications for their use during breastfeeding [26].

MW 314, pKat 8.2; pKa2 2.3, tmax 1-3 h, Vd 1.6-2.4 L/ kg, EPB 15%, t_0,5_ elimin. 2-3 h, BA oral 50%, M/P 1.96.7, RID 2.53-9.14% [2].

### Medications used during a routine termination of general anesthesia

#### Neostigmine (pregnancy: C, lactation: no data available)

A cholinergic drug used in the treatment of *myasthenia gravis*. There is no description of a teratogenic influence on the fetus. There is no data available about the permeation of the drug into mother’s milk. A single dose in the perioperative period in the treatment of intestinal atony or the bladder is of no significance for breastfeeding.

MW 223 g/mol (334 g/mol as methylsulfate), EPB 15-25%, t_0,5_ elimin. 50-90 minutes, BA oral 1-3% [24].

#### Atropine (pregnancy: C, lactation: L3)

A belladonna alkaloid. A classical parasympatholytic drug which blocks the effect of acetylcholine through the blockage of muscarinic receptors. It is easily distributed throughout the whole organism. After a few minutes since application, it reaches a concentration in the fetal blood similar to that in the mother’s blood concentration. It affects the activity of the fetal heart and respiratory activity. There has not been any reported developmental anomaly or toxicity for the fetus. It permeates into the mother’s blood. The low absorption by the baby in combination with an individual reaction to the drug indicates the necessity of caution in its usage for breastfeeding mothers. Generally, atropine should be avoided, but it is not evidently contraindicated in pregnant and breastfeeding mothers [22, 23].

MW 289, pKa 9.8, tmax 1h, Vd 23-3.6 L/kg, EPB 14-22%, t_0,5_ elimin. 3-4 h, PHL 6.9 h (< 2 years), BA oral 90% *[2*, 24].

## Agents that contract the uterus

### Oxytocin (pregnancy: C, lactation: L2)

This is the endogenic hormone octapeptide released into the blood through the posterior pituitary gland. It induces a contracting effect on the uterus muscle and myoepithelial cells and a vasopressin and antidiuretic effect. Excessive stimulation of the uterus muscle can lead to too much tension in the uterus, disorders of placenta perfusion, hypoxia or even death of the fetus. Oxytocin is neutralized by specific oxytocinases in the liver, spleen and the ovaries. During pregnancy, they are activated by other enzymes produced in the placenta. The half-life duration of oxytocin is very short.

The synthetic preparation is administered intravenously or through the nose. Oxytocin in a minor concentration is secreted into mother’s milk. The concentration in mother s milk at postpartum on the 1st to the 5th day was respectively 4.5; 4.7; 4.0; 3.2 and 3.3 microunits/mL [27]. The absorption through the gastrointestinal tract of the baby is minimal. After oral application, oxytocin is digested by chymotrypsin and metabolized by the liver.

Oxytocin is sometimes used by breastfeeding mothers to trigger the outflow of milk from the calciferous gland. It does not increase the production of milk.

There is no observable toxic effect of the drug on the neonate. Oxytocin used through the nose for a prolonged time by a breastfeeding mother can lead to the addiction of the newborn and should be limited to no more than one week after birth.

MW >1000 g/mol, pKa 6.56, t0,5 3-5 minutes, BA oral - minimal [2].

RECOMMENDATION: Oxytocin may be used to induce labor and to lead the process. However, it should be used with special caution and only with monitoring the contraction activity of the uterus and fetal heard activity.

### Carbetocin (pregnancy: C, lactation: no data available)

A synthetic oxytocin analogue that works for a long time. Carbetocin is used directly after a planned cesarean section, in which local or spinal anesthesia was used. When compared to oxytocin, binding to its receptor takes a longer time. Carbetocin is administered to the mother immediately after birth in order to prevent atonia of the uterus. Thanks to using carbetocin we can avoid repeating doses of oxytocin and thereby its compounding adverse effects. The bioavailability after intramuscular administration is 80% and the mean half-life is 40 minutes. An efficient dose of 100 mcg administered once intravenously or intramuscularly generates the same effect as a 16-hour constant infusion of oxytocin.

**Table I:** Summary of the pharmacologic characteristics of analgesics [2]. Tabela I. Podsumowanie właściwości farmakologicznych leków przeciwbólowych [2].

Medication *Lek*	t_0,5_ [h]	PHL [h]	t_max_	MW [g/mol]	Vd [L/kg]	M/P	EPB [%]	BA oral [%]	pKa	RID [%]
Paracetamol	1-3	1-3	10-60 minutes	151	0.8-1.0	0.91-1.42	10-25	>85	9.5	6.41-24.23
Nalbufine	5	0.86	2-15 minutes (IV, IM)	357	2.4-7.3	1.2	50	16	8.71 9.96	0.5-0.8
Ibuprofen	1.8-2.5		1-2 h	206	0.14		>99	80	4.4	0.1-0.7

Carbetocin permeates into milk in a very small concentration. The maximum concentration in mother’s milk was 56 times smaller than in the plasma in the 120th minute.

It is considered that small amounts of carbetocin which permeate after a single injection into colostrum or mother’s milk, and are then swallowed by the baby, go through an enzymatic degradation in the bowel. That is why breastfeeding is possible directly after birth with no limitations.

During clinical studies, there were no reports about a significant effect that blocks lactation.

MW 988 g/mol, t_max_ 15-30 minutes, t_0,5_ 29-53 minutes, RID 0.27-0.29% [2].

## Analgesia after CC

### Paracetamol (pregnancy: B, lactation: LI)

A minor portion of the drug permeates into the mother’s blood. Its concentration in breast milk is low. The amount of the drug available to the newborn is from 0.04 to 0.23% of the mother’s dose [281. Although the drug’s metabolism and kidney excretion in newborns are undeveloped, no accumulation of the drug has been observed, even during extended use.

Paracetamol is considered compatible with breastfeeding and is a drug of choice during pregnancy and breastfeeding. However, it is important to know that some human trials reveal a correlation between paracetamol use in pregnant mothers and an increased risk of wheezing, allergic rhinitis and asthma in children later in life [29, 30]. A similar connection (wheezing) was found in breastfed infants whose mothers have taken paracetamol [31] but these results were refuted, because the study was short-term and based on too small a group (n=11). More research is necessary.

RECOMMENDATION: Paracetamol is permissible during breastfeeding [32]. Apart from ibuprofen, paracetamol belongs to the analgetics of choice for breastfeeding women.

### Nalbufine (pregnancy: B, lactation: L2)

A painkiller with a strong effect similar to morphine. It is both an agonist and antagonist of the opioid receptor. The M/ P ratio of nalbufine is about 1 [23]. The amount of medicine that the breastfed baby receives is 0.012% of the mother’s dose.

RECOMMENDATION: A medication recommended for breastfeeding mothers [32].

### Ibuprofen (pregnancy: C and D (≥30 week of gestation), lactation: L1)

A non-steroidal anti-inflammatory painkiller. It is frequently used in children. Less than 0.6% of the dose permeates into mother’s milk. After administration of 800-1600 mg daily, the concentration of the medication in mother’s milk was not detected [23]. There have been no reported adverse effects for neonates being breastfed by mothers who have taken ibuprofen [23].

RECOMMENDATION: Ibuprofen is a medication of choice for breastfeeding mothers (Table I).

## Local anesthetic drugs used for conducting anesthesia

### Bupivacaine (Marcaine spinal heavy 0.5%) (pregnancy: C, lactation: L2)

A medication frequently used for conducting anesthesia. The concentration of bupivacaine in milk 2-48 hours after application is untraceable. The drug is considered safe for newborns being breastfed. There have been no reports of adverse effects for the baby [2].

### Lidocaine (pregnancy: B, lactation: L2)

A medication used for local anesthesia. It is excreted into breast milk with a RID value of 0.5-3.1 %. After the local application of 183 mg to 27 women, the concentration of lidocaine in milk after 2, 6 and 12 hours was 0.09, 0.06 and 0.04 mg/ L, respectively. Considering the dose, route and concentration in the mother’s plasma, it is unlikely the neonate would ingest clinically relevant amounts [28].

RECOMMENDATION: Mothers who receive lidocaine for local analgesia can safely breastfeed (Table II).

### Ropivacaine (pregnancy: B, lactation: L2)

A 1% solution for subarachnoid use, 0.75% for perineural and epidural application. In comparison with bupivacaine, it exhibits a weaker depressive effect on the cardiovascular system. At the same time the labor pain mitigating effect is stronger. It permeates through the placenta, and in the cord blood it reaches a concentration of 30% of that of the mother. The effect on the fetus and the breastfed newborn is mild and temporary.

**Table II:** Summary of pharmacologic characteristics of local anesthetics [2]. Tabela II. Podsumowanie właściwości farmakologicznych leków miejscowo znieczulających [2].

Medication *Lek*	t[0h,5]	PHL [h]	t_max_	MW [g/mol]	V. [L/kg]	M/P	EPB [%]	BA oral [%]	pKa	RID [%]
0.5% Bupivacaine *0,5% bupiwakaina*	2.7	8.1	30-45 minutes	288	0.4-1.0	0.37	95		8.1	0.85-2.92
*2%* Lidocaine *2% lidokaina*	1.8	3	Immediately (IM, IV) *natychmiast* (IM, IV)	234	1.3	0.4	70	<35	7.9	0.5-3.1

In the Matsota (2009) trial, all the 25 infants whose mothers were administered ropivacaine with fentanyl (epidural analgesia after CS) had normal Apgar and Neurological Capacity Scores. After 24 hours since labor the concentration of the medication in the newborns blood was untraceable. There was no research on the topic of distant results concerning the development of these babies [33].

MW 328, pKa 8.07, tmax 43 minutes (epidural), Vd 0.58 L/kg, EPB 94%, t_0,5_ 4.2 h (epidural), RID 0.5-3.1 [33].

## Medications that influence coagulation

### Tranexamic acid (pregnancy: B, lactation: L3)

Efficient medication with a quick effect. It is used in the case of bleeding caused by excessive fibrinolysis. It blocks the activity of plasmin. Its tmax is 3 hours. Only 3% of the drug binds to proteins, mainly to plasminogen. After administering to a pregnant woman, the concentration in the cord blood reaches 40-60% and 90% of the dose is excreted unchanged in the urine within 24 hours.

The concentration in the milk of a breastfeeding mother reaches 1% of value in the mother’s blood. Its high percentage of permeation into the mother’s milk means that breastfeeding is not recommended during treatment.

MW 157 g/mol, pKa 4.77, tmax 3 h, BA oral 45%, V_d_ 0.39 L/kg, EPB 3%, t_0,5_ elim. 11 h [2].

## Preparations containing coagulation factors

### Eptacog alfa, activated (pregnancy: C, lactation: L2)

A recombinan t human Vila factor of coagulation (rFVIIa). A very high particle mass (50 000 D) disenables the permeation into mother’s milk.

MW 50 000 Daltons, Vd 0.13-0.165 L/kg, t0.5 elim. 3.9-6 h, PHL 1*.7-*2.7 h [34].

## Summary

1. The neonatologist should be informed about all the medications used during obstetric anesthesia.
2. Because of the pharmacodynamic and pharmacokinetic differences described, it is possible and necessary to choose the medication with the most favorable profile to reach the optimal effect.
3. Each neonate after cesarean section should be observed in terms of adverse drug reaction, mainly from the respiratory and circulatory systems.
4. When the termination of breastfeeding is being considered, it is important to take into account the risk for the baby connected to administering an artificial formula and the negative impact of prohibiting breastfeeding on the mother.

## Abbreviations Used

BA oral: Oral bioavailability
C: Plasma or milk concentrations at peak
CNS: Central nervous system
CS: Cesarean section
EPB: The extent of protein binding
h: Hour
IM: Intramuscular
IN: Intranasal
IV: Intravenous
M/P: Milk/plasma ratio
MW: Molecular weight
PHL: Pediatric elimination half-life
pKa: pH on which the drug is equallyionicand nonionic
RID: Relative Infant Dose
t_0,5_: Half-life
_t0,5_ distrib.: Adult distribution half-life
t0,5 redistrib.: Adult redistribution half-life
t _0,5_ elim.: Adult elimination half-life
t _max_: Time to peak plasma level
V, _d_: Volume of distribution
